# High-quality permanent draft genome sequence of *Rhizobium sullae* strain WSM1592; a *Hedysarum coronarium* microsymbiont from Sassari, Italy

**DOI:** 10.1186/s40793-015-0020-2

**Published:** 2015-07-24

**Authors:** Ron Yates, John Howieson, Sofie E. De Meyer, Rui Tian, Rekha Seshadri, Amrita Pati, Tanja Woyke, Victor Markowitz, Natalia Ivanova, Nikos Kyrpides, Angelo Loi, Brad Nutt, Giovanni Garau, Leonardo Sulas, Wayne Reeve

**Affiliations:** Department of Agriculture and Food, Western Australia, Australia; Centre for Rhizobium Studies, Murdoch University, Murdoch, Western Australia Australia; DOE Joint Genome Institute, Walnut, Creek, CA USA; Biological Data Management and Technology Center, Lawrence Berkeley National Laboratory, Berkeley, CA USA; Department of Biological Sciences, Faculty of Science, King Abdulaziz University, Jeddah, Saudi Arabia; Department of Agriculture, University of Sassari, Sardinia, Italy; Institute for the Animal Production System in the Mediterranean Environment (ISPAAM), National Research Council (CNR), Sassari, Italy

**Keywords:** Root-nodule bacteria, Nitrogen fixation, Rhizobia, *Alphaproteobacteria*, GEBA-RNB

## Abstract

*Rhizobium sullae* strain WSM1592 is an aerobic, Gram-negative, non-spore-forming rod that was isolated from an effective nitrogen (N_2_) fixing root nodule formed on the short-lived perennial legume *Hedysarum coronarium* (also known as *Sulla coronaria* or Sulla). WSM1592 was isolated from a nodule recovered from *H. coronarium* roots located in Ottava, bordering Sassari, Sardinia in 1995. WSM1592 is highly effective at fixing nitrogen with *H. coronarium*, and is currently the commercial Sulla inoculant strain in Australia. Here we describe the features of R. sullae strain WSM1592, together with genome sequence information and its annotation. The 7,530,820 bp high-quality permanent draft genome is arranged into 118 scaffolds of 118 contigs containing 7.453 protein-coding genes and 73 RNA-only encoding genes. This rhizobial genome is sequenced as part of the DOE Joint Genome Institute 2010 Genomic Encyclopedia for Bacteria and Archaea-Root Nodule Bacteria (GEBA-RNB) project.

**Electronic supplementary material:**

The online version of this article (doi:10.1186/s40793-015-0020-2) contains supplementary material, which is available to authorized users.

## Introduction

The accessibility and supply of nitrogen fertilizer is an ever-increasing challenge that world agriculture faces [1]. Despite the fact the Earth’s atmosphere consists of approximately 78 % dinitrogen, it is in a form that must be converted before it can be utilised by plants [2]. Conversion of N_2_ can be achieved by the chemical synthesis of natural gas but these methods can be considered unsustainable because of the use of exhaustible and costly fossil fuel resources [3]. In addition, the manufacturing process not only increases the greenhouse gas emissions but also field N fertiliser application have been directly linked to contaminating and leading to detrimental effects in ecosystems and waterways. Alternatively, a more sustainable and environmentally friendly process of acquiring N is through the biological process of N fixation by diazotrophs [2]. Most biological fixation in world agriculture is provided from the process of symbiotic nitrogen fixation, which occurs following the successful formation of an effective symbiosis by leguminous plants and bacterial microsymbionts [4].

The productive efficiencies of SNF in agricultural areas rely on considerable efforts by researchers and producers in matching suitable legume hosts with their compatible microsymbionts [5]. Some agricultural areas farm with indigenous legumes, while others embark on introducing exotic legumes and their compatible microsymbionts from different geographical locations that are edaphically and climatically suited to their own [4]. In Australia for instance, selection programs have enabled the domestication of new Mediterranean legume species and their microsymbionts [6]. One such grazing legume species commercially introduced into Australian and New Zealand agriculture includes the Papilionoid legume *Hedysarum coronarium* (also known as *Sulla coronaria* or Sulla). Sulla is a deep-rooted, short-lived perennial pasture legume that is grown throughout Mediterranean countries where it is fed green, used for silage or as hay [7]. It is noted that the microsymbionts of Sulla display a high level of specificity for nodulation and nitrogen fixation [8]. However, when effectively nodulated Sulla plants have the ability to biologically fix large amounts of nitrogen for increased paddock fertility [9].

*Rhizobium sullae* strain WSM1592 is the current Australian commercial inoculant for Sulla after replacing strain CC1335 in 2006. This strain has also been deposited in the Western Australian Soil Microbiology collection and is available for research. WSM1592 was isolated in 1995 from a nodule collected from a Sulla plant sampled on a roadside in calcareous loamy sand near the Ottava agriculture research farm, east of Sassari in Sardinia, Italy. The location has a Mediterranean climate with a long-term mean seasonal rainfall of 547 mm. Here we present a preliminary description of the general features for *Rhizobium sullae* strain WSM1592 together with its genome sequence and annotation.

## Organism information

### Classification and features

**Fig. 1:**
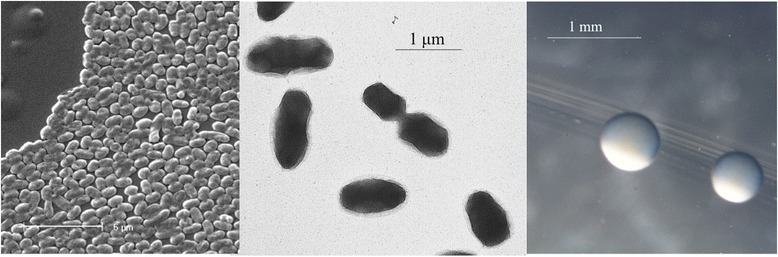
Images of *Rhizobium sullae* strain WSM1592 using scanning (Left) and transmission (Center) electron microscopy and the appearance of colony morphology on solid media (Right)

*R. sullae* strain WSM1592 is a motile, Gram-negative rod (Fig. 1 Left and Center) in the order *Rhizobiales* of the class *Alphaproteobacteria*. It is fast growing, forming colonies within 3–4 days when grown on half strength Lupin Agar (½LA) [10], tryptone-yeast extract agar (TY) [11] or a modified yeast-mannitol agar (YMA) [12] at 28 °C. Colonies on ½LA are white-opaque, slightly domed and moderately mucoid with smooth margins (Fig. 1 Right).

**Fig. 2:**
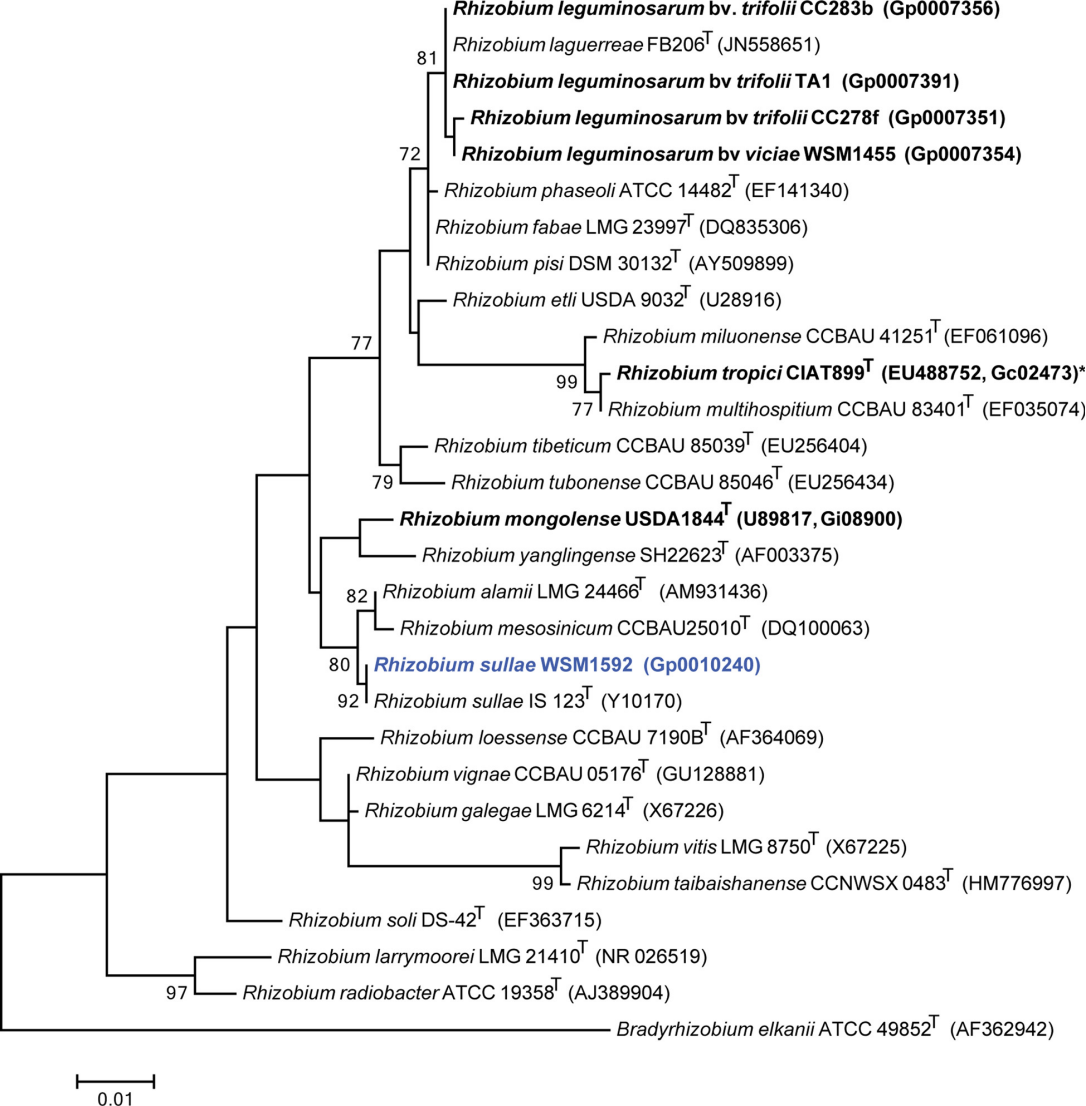
Phylogenetic tree highlighting the position of *R. sullae* strain WSM1592 (shown in blue print) relative to other type and non-type rhizobia strains using a 901 bp internal region of the 16S rRNA gene. Bradyrhizobium elkanii ATCC 49852^T^ was used as an outgroup. All sites were informative and there were no gap-containing sites. Phylogenetic analyses were performed using MEGA, version 5.05 [33]. The tree was built using the maximum likelihood method with the General Time Reversible model. Bootstrap analysis with 500 replicates was performed to assess the support of the clusters. Type strains are indicated with a superscript T. Strains with a genome sequencing project registered in GOLD [18] have the GOLD ID mentioned after the strain number and represented in bold, otherwise the NCBI accession number is provided. Finished genomes are designated with an asterisk

Figure 2 shows the phylogenetic relationship of *R. sullae* strain WSM1592 in a 16S rRNA gene sequence based tree. This strain is phylogenetically the most related to *Rhizobium sullae* IS 123^T^, *Rhizobium leguminosarum*USDA 2370^T^ and *Rhizobium phaseoli*ATCC 14482^T^ with sequence identities to the WSM1592 16S rRNA gene sequence of 100 %, 99.84 % and 99.84 %, respectively, as determined using the EzTaxon-e server [13]. *Rhizobium sullae* IS 123^T^ was isolated from a *Hedysarum coronarium* root nodule discovered in Southern Spain [14]. In contrast, *R. leguminosarum*USDA 2370^T^ was isolated from an effective nodule of *Pisum sativum* and is also able to nodulate *Trifolium repens* and *Phaseolus vulgaris* [15]. *R. phaseoli*ATCC 14482^T^ was originally isolated from nodules of Phaseolus vulgaris and has been shown to nodulate *Trifolium repens*, but not *Pisum sativum* [15].

Minimum Information about the Genome Sequence [16] of WSM1592 is provided in Table 1 and Additional file 1: Table S1.

**Table 1:** Classification and general features of *R. sullae* strain WSM1592 [16, 34]

MIGS ID	Property	Term	Evidence code
	Classification	Domain Bacteria	TAS [35]
		Phylum *Proteobacteria*	TAS [36, 37]
		Class *Alphaproteobacteria*	TAS [38]
		Order *Rhizobiales*	TAS [39]
		Family *Rhizobiaceae*	TAS [40]
		Genus *Rhizobium*	TAS [41]
		Species *Rhizobium sullae*	TAS [14]
		(Type) *strain WSM1592*	IDA
	Gram stain	Negative	IDA
	Cell shape	Rod	IDA
	Motility	Motile	IDA
	Sporulation	Non-sporulating	NAS
	Temperature range	Not reported	
	Optimum temperature	28 °C	NAS
	pH range; Optimum	Not reported	
	Carbon source	Not reported	
MIGS-6	Habitat	Soil, root nodule, on host	IDA
MIGS-6.3	Salinity	Non-halophile	NAS
MIGS-22	Oxygen requirement	Aerobic	IDA
MIGS-15	Biotic relationship	Free living, symbiotic	IDA
MIGS-14	Pathogenicity	Non-pathogenic	NAS
MIGS-4	Geographic location	Sassari, Italy	IDA
MIGS-5	Soil collection date	20 May 1995	IDA
MIGS-4.1	Latitude	8.465	IDA
MIGS-4.2	Longitude	40.777	IDA
MIGS-4.4	Altitude	83 m	IDA

Evidence codes – *IDA* Inferred from Direct Assay; *TAS* Traceable Author Statement (i.e., a direct report exists in the literature); *NAS* Non-traceable Author Statement (i.e., not directly observed for the living, isolated sample, but based on a generally accepted property for the species, or anecdotal evidence). These evidence codes are from the Gene Ontology project [42]

#### Symbiotaxonomy

*Hedysarum coronarium* is a short-lived perennial pasture legume native to the Mediterranean basin and throughout the *Hedysarum* genus there is a large degree of specificity in symbiotic compatibility within this region [8]. *Rhizobium sullae*WSM1592 nodulates (Nod+) and fixes nitrogen effectively (Fix+) with *Hedysarum coronarium**.* However, inoculation of *H. spinosissimum*, *H. flexuosum* and *H. carnosum* with WSM1592 results in mostly Nod- but always Fix-.

## Genome sequencing information

### Genome project history

This organism was selected for sequencing on the basis of its environmental and agricultural relevance to issues in global carbon cycling, alternative energy production, and biogeochemical importance, and is part of the *Genomic Encyclopedia of Bacteria and Archaea*, The Root Nodulating Bacteria chapter project at the U.S. Department of Energy, Joint Genome Institute [17]. The genome project is deposited in the Genomes OnLine Database [18] and the high-quality permanent draft genome sequence in IMG [19]. Sequencing, finishing and annotation were performed by the JGI using state of the art sequencing technology [20]. A summary of the project information is shown in Table 2.

**Table 2:** Project information

MIGS ID	Property	Term
MIGS-31	Finishing quality	High-quality permanent draft
MIGS-28	Libraries used	Illumina Std PE (2x150bps)
MIGS-29	Sequencing platforms	Illumina HiSeq 2000
MIGS-31.2	Fold coverage	877x
MIGS-30	Assemblers	Velvet 1.1.04; Allpaths-LG r39750
MIGS-32	Gene calling methods	Prodigal 1.4
	Locus Tag	A3C1
	Genbank ID	ATZB00000000
	Genbank Date of Release	December 12, 2013
	GOLD ID	Gp0010240
	BIOPROJECT	PRJNA165333
MIGS-13	Source Material Identifier	WSM1592
	Project relevance	Symbiotic N_2_ fixation, agriculture

#### Growth conditions and genomic DNA preparation

*R. sullae*WSM1592 was cultured to mid logarithmic phase in 60 ml of TY rich media [11] on a gyratory shaker at 28 °C. DNA was isolated from the cells using a CTAB (Cetyl trimethyl ammonium bromide) bacterial genomic DNA isolation method [21].

### Genome sequencing and assembly

The draft genome of *R. sullae* strain WSM1592 was generated at the DOE Joint Genome Institute using state of the art technology [20]. An Illumina Std shotgun library was constructed and sequenced using the Illumina HiSeq 2000 platform which generated 29,255,624 reads. All general aspects of library construction and sequencing performed at the JGI can be found at the JGI’s web site [22]. All raw Illumina sequence data was passed through DUK, a filtering program developed at JGI, which removes known Illumina sequencing and library preparation artifacts (Mingkun L, Copeland A, Han J. unpublished). Following steps were then performed for assembly: (1) filtered Illumina reads were assembled using Velvet (version 1.1.04) [23] (2) 1–3 Kbp simulated paired end reads were created from Velvet contigs using wgsim [24] (3) Illumina reads were assembled with simulated read pairs using Allpaths–LG (version r39750) [25]. Parameters for assembly steps were: 1) Velvet (velveth: 63 –shortPaired and velvetg: −very_clean yes –exportFiltered yes –min_contig_lgth 500 –scaffolding no–cov_cutoff 10) 2) wgsim (−e 0 –1 76 –2 76 –r 0 –R 0 –X 0) 3) Allpaths–LG (PrepareAllpathsInputs: PHRED_64 = 1 PLOIDY = 1 FRAG_COVERAGE = 125 JUMP_COVERAGE = 25 LONG_JUMP_COV = 50, RunAllpathsLG: THREADS = 8 RUN = std_shredpairs TARGETS = standard VAPI_WARN_ONLY = True OVERWRITE = True). The final draft assembly contained 118 contigs in 118 scaffolds. The total size of the genome is 7.5 Mbp and the final assembly is based on 2,498,075,850 bp of Illumina data, which provides an average of 877× coverage of the genome.

### Genome annotation

Genes were identified using Prodigal [26], as part of the DOE-JGI genome annotation pipeline [27, 28]. The predicted CDSs were translated and used to search the National Centre for Biotechnology Information non-redundant database, UniProt, TIGRFam, Pfam, KEGG, COG, and InterPro databases. The tRNAScanSE tool [29] was used to find tRNA genes, whereas ribosomal RNA genes were found by searches against models of the ribosomal RNA genes built from SILVA [30]. Other non–coding RNAs such as the RNA components of the protein secretion complex and the RNase P were identified by searching the genome for the corresponding Rfam profiles using INFERNAL [31]. Additional gene prediction analysis and manual functional annotation was performed within the Integrated Microbial Genomes-Expert Review system [32] developed by the Joint Genome Institute, Walnut Creek, CA, USA.

## Genome properties

The genome is 7,530,820 nucleotides 59.87 % GC content (Table 3 and comprised of 118 scaffolds of 118 contigs. From a total of 7,526 genes, 7,453 were protein encoding and 73 RNA only encoding genes. The majority of genes (78.42 %) were assigned a putative function whilst the remaining genes were annotated as hypothetical. The distribution of genes into COG functional categories is presented in Table 4.

**Table 3:** Genome statistics

Attribute	Value	% of Total
Genome size (bp)	7,530,820	100.00
DNA coding (bp)	6,571,312	87.26
DNA G + C (bp)	4,508,646	59.87
DNA scaffolds	118	
Total genes	7,526	100.00
Protein coding genes	7,453	99.03
RNA genes	73	0.97
Pseudo genes	0	0
Genes in internal clusters	538	7.15
Genes with function prediction	5,902	78.42
Genes assigned to COGs	5,148	68.40
Genes with Pfam domains	6,174	82.04
Genes with signal peptides	659	8.76
Genes with transmembrane helices	1,699	22.58
CRISPR repeats	0	0

**Table 4:** Number of genes associated with the general COG functional categories

Code	Value	% age	COG Category
J	186	3.25	Translation, ribosomal structure and biogenesis
A	0	0.00	RNA processing and modification
K	554	9.67	Transcription
L	158	2.76	Replication, recombination and repair
B	2	0.03	Chromatin structure and dynamics
D	36	0.63	Cell cycle control, Cell division, chromosome partitioning
V	65	1.13	Defense mechanisms
T	211	3.68	Signal transduction mechanisms
M	293	5.11	Cell wall/membrane/envelope biogenesis
N	68	1.19	Cell motility
U	112	1.95	Intracellular trafficking, secretion, and vesicular transport
O	167	2.91	Posttranslational modification, protein turnover, chaperones
C	327	5.71	Energy production and conversion
G	614	10.71	Carbohydrate transport and metabolism
E	684	11.94	Amino acid transport and metabolism
F	107	1.87	Nucleotide transport and metabolism
H	174	3.04	Coenzyme transport and metabolism
I	196	3.42	Lipid transport and metabolism
P	318	5.55	Inorganic ion transport and metabolism
Q	138	2.41	Secondary metabolite biosynthesis, transport and catabolism
R	743	12.96	General function prediction only
S	578	10.09	Function unknown
-	2378	31.60	Not in COGS

The total is based on the total number of protein coding genes in the genome

## Conclusions

*Rhizobium sullae*WSM1592 was isolated from a root nodule of *Hedysarum coronarium* (also known as *Sulla coronaria*). Phylogenetic analysis revealed that WSM1592 is the most closely related to *Hedysarum coronarium* IS 123^T^, which was also isolated from Hedysarum coronarium growing in Southern Spain. The genome of WSM1592 is the first to be described for a strain of *Rhizobium sullae* and is 7.5 Mbp, with a GC content of 59.87 %. As expected this genome contains the nitrogenase-RXN MetaCyc pathway characterized by the multiprotein nitrogenase complex and has been shown to fix effectively with *Hedysarum coronarium*. The genome attributes of WSM1592 will be important for the characterisation of the genetic determinants required for the establishment of an effective symbiosis with *Hedysarum*.

## Additional file

Additional file 1: Table S1. Associated MIGS record for WSM1592.

## Abbreviations

GEBA-RNB: *Genomic Encyclopedia of Bacteria and Archaea* – *Root Nodule Bacteria*
JGI: Joint Genome Institute
TY: Trypton Yeast
CTAB: Cetyl trimethyl ammonium bromide
WSM: Western Australian Soil Microbiology
SNF: Symbiotic Nitrogen Fixation
