# Supplementary material for: High-quality permanent draft genome sequence of Rhizobium sullae strain WSM1592; a Hedysarum coronarium microsymbiont from Sassari, Italy
Source: Stand Genomic Sci. 2015 Jul 24;10:44. doi: 10.1186/s40793-015-0020-2 (PMC4572446; doi:10.1186/s40793-015-0020-2)
Supplement: Additional file 1: Table S1. — Associated MIGS record for WSM1592. [file 40793_2015_20_MOESM1_ESM.pdf]

Table S1. Associated MIGS record for WSM1592

MIGS-ID	Field name	Description
**MIGS-1**	Submit to INSDC/Trace archives	
**1.1**	PID	
**1.2**	Trace Archive	
**MIGS-2**	MIGS CHECK LIST TYPE	
**MIGS-3**	Project Name	GEBA - Root Nodulating
**MIGS-4**	Geographic Location	Sardinia
**4.1**	Latitude	40.777
**4.2**	Longitude	8.465
**4.3**	Depth	10-20 cm
**4.4**	Altitude	80m
**MIGS-5**	Time of Sample collection	
**MIGS-6**	Habitat (EnvO)	Ottava University farm
**6.1**	Temperature	28
**6.2**	pH	5-8
**6.3**	Salinity	
**6.4**	Chlorophyll	
**6.5**	Conductivity	
**6.6**	Light intensity	
**6.7**	Dissolved organic carbon (DOC)	
**6.8**	Current	
**6.9**	Atmospheric data	
**6.10**	Density	
**6.11**	Alkalinity	
**6.12**	Dissolved oxygen	
**6.13**	Particulate organic carbon (POC)	
**6.14**	Phosphate	
**6.15**	Nitrate	
**6.16**	Sulfates	
**6.17**	Sulfides	
**6.18**	Primary production	
**MIGS-7**	Subspecific genetic lineage	
**MIGS-9**	Number of replicons	
**MIGS-10**	Extrachromosomal elements	
**MIGS-11**	Estimated Size	7.5 Mbp
**MIGS-12**	Reference for biomaterial or Genome report	
**MIGS-14**	Known Pathogenicity	Non-pathogen
**MIGS-15**	Biotic Relationship	Symbiotic
**MIGS-16**	Specific Host	*Hedysarum coronarium*
**MIGS-17**	Host specificity or range (taxid)	
**MIGS-18**	Health status of Host	
**MIGS-19**	Trophic Level	
**MIGS-22**	Relationship to Oxygen	Aerobe
**MIGS-23**	Isolation and Growth conditions	TY medium [11], 28°C,
**MIGS-27**	Nucleic acid preparation	CTAB
**28.1**	Library size	
**28.2**	Number of reads	29,255,624
**28.3**	Vector	
**MIGS-29**	Sequencing method	Illumina HiSeq 2000
**MIGS-30**	Assembly	
**30.1**	Assembly method	Velvet version 1.1.04,
**30.2**	Estimated error rate	
**30.3**	Method of calculation	
**MIGS-31**	Finishing strategy	
**31.1**	Status	High-quality permanent draft
**31.2**	Coverage	877x
**31.3**	Contigs	118
**MIGS-32**	Relevant SOPs	
**MIGS-33**	Relevant e-resources	
